# RETRACTION: MicroRNA‐224 Regulates Self‐Renewal of Mouse Spermatogonial Stem Cells via Targeting DMRT1

**DOI:** 10.1111/jcmm.71256

**Published:** 2026-06-23

**RETRACTION**: N. Cui , G. Hao , Z. Zhao , F. Wang , J. Cao , and A. Yang , “MicroRNA‐224 Regulates Self‐Renewal of Mouse Spermatogonial Stem Cells via Targeting DMRT1,” Journal of Cellular and Molecular Medicine 20, no. 8 (2016): 1503–1512, 10.1111/jcmm.12838.27099200 PMC4956939

The above article, published online on 21 April 2016 in Wiley Online Library (wileyonlinelibrary.com), has been retracted by agreement between the journal Editor‐in‐Chief, Stefan N. Constantinescu; the Foundation for Cellular and Molecular Medicine; and John Wiley & Sons Ltd.

Following publication, concerns were raised by third parties regarding overlap between Figure 2C in this article and unrelated figures in previously published articles [1, 2, 3]. The authors did not respond to requests for an explanation or provide the original data. The retraction has been agreed because these concerns affect the interpretation of the data and results presented, and the conclusions of this manuscript have to be considered insufficiently supported.

Author N. Cui was informed of the decision to retract. Authors G. Hao, Z. Zhao, F. Wang, J. Cao, and A. Yang could not be reached.

## References

[1] Z. Xia , F. Liu , J. Zhang , and L. Liu , “Decreased Expression of miRNA‐204‐5p Contributes to Glioma Progression and Promotes Glioma Cell Growth, Migration and Invasion,” PLOS One 10, no. 7 (2015): e0132399, 10.1371/journal.pone.0132399. 26134825 PMC4489611

[2] J. Ma , J. Liu , C. Lu , and D. Cai , “RETRACTED ARTICLE: Pachymic Acid Induces Apoptosis via Activating ROS‐Dependent JNK and ER Stress Pathways in Lung Cancer Cells,” Cancer Cell International 15, no. 78 (2015): 78, 10.1186/s12935-015-0230-0. 26244039 PMC4524283

[3] C. Wang , X. Wang , Z. Su , H. Fei , X. Liu , and Q. Pan , “miR25 Promotes Hepatocellular Carcinoma Cell Growth Migration and Invasion by Inhibiting RhoGDI1,” Oncotarget 6 (2015): 36231–36244, 10.18632/oncotarget.4740. 26460549 PMC4742173

